# Human induced pluripotent stem cell derived neurons as a model for Williams-Beuren syndrome

**DOI:** 10.1186/s13041-015-0168-0

**Published:** 2015-11-24

**Authors:** Shahryar Khattak, Elise Brimble, Wenbo Zhang, Kirill Zaslavsky, Emma Strong, P. Joel Ross, Jason Hendry, Seema Mital, Michael W. Salter, Lucy R. Osborne, James Ellis

**Affiliations:** Program in Developmental and Stem Cell Biology, Hospital for Sick Children, Toronto, ON Canada; Department of Molecular Genetics, University of Toronto, Toronto, ON Canada; Program in Neurosciences & Mental Health, Hospital for Sick Children, Toronto, ON Canada; Department of Physiology, University of Toronto, Toronto, ON Canada; University of Toronto Centre for the Study of Pain, University of Toronto, Toronto, ON Canada; Institute of Medical Science, University of Toronto, Toronto, ON Canada; Department of Pediatrics, Hospital for Sick Children, Toronto, ON Canada; Department of Medicine, University of Toronto, Toronto, ON Canada; Developmental and Stem Cell Biology, The Hospital for Sick Children, Peter Gilgan Centre for Research and Learning, 686 Bay St, 16th Floor - Room 9705, Toronto, ON M5G 0A4 Canada

**Keywords:** Williams-Beuren Syndrome, Human pluripotent stem cell, Neural differentiation, Transcriptome, Disease modeling, Ion channel

## Abstract

**Background:**

Williams-Beuren Syndrome (WBS) is caused by the microdeletion of approximately 25 genes on chromosome 7q11.23, and is characterized by a spectrum of cognitive and behavioural features.

**Results:**

We generated cortical neurons from a WBS individual and unaffected (WT) control by directed differentiation of induced pluripotent stem cells (iPSCs). Single cell mRNA analyses and immunostaining demonstrated very efficient production of differentiated cells expressing markers of mature neurons of mixed subtypes and from multiple cortical layers. We found that there was a profound alteration in action potentials, with significantly prolonged WBS repolarization times and a WBS deficit in voltage-activated K^+^ currents. Miniature excitatory synaptic currents were normal, indicating that unitary excitatory synaptic transmission was not altered. Gene expression profiling identified 136 negatively enriched gene sets in WBS compared to WT neurons including gene sets involved in neurotransmitter receptor activity, synaptic assembly, and potassium channel complexes.

**Conclusions:**

Our findings provide insight into gene dysregulation and electrophysiological defects in WBS patient neurons.

**Electronic supplementary material:**

The online version of this article (doi:10.1186/s13041-015-0168-0) contains supplementary material, which is available to authorized users.

## Background

Williams-Beuren Syndrome (WBS; OMIM #194050) is a rare neurodevelopmental disorder caused by the hemizygous deletion of a cluster of ~25 protein coding genes at 7q11.23. It is characterized by cardiac abnormalities, including supravalvular aortic stenosis, infantile hypercalcemia, characteristic facial features and a unique constellation of cognitive and behavioural impairments [1, 2]. Specifically, individuals with WBS have relatively preserved expressive language skills, but extreme weakness in visuospatial construction alongside over-friendliness, social disinhibition, and a high prevalence of anxiety-related disorders [3, 4]. This syndrome provides a unique opportunity to study the molecular basis of complex behavioural and neurological phenotypes in relation to a well characterized genetic lesion. Thus far, functional and structural imaging studies have implicated specific brain regions in altered visuospatial processing [5] and facial recognition [6], but, how the deleted genes contribute to this altered function, or to the development of more common psychiatric features associated with WBS remains unclear.

Both animal model and human studies have been used to attempt to understand the molecular basis for the WBS neurological features, but both approaches have inherent limitations. Animal models have offered critical insight into the cellular and molecular bases of *in vivo* phenotypes [7], but in a non-human context, and so require validation in relevant human cells. The study of human tissue samples has explored the effect of 7q11.23 dosage on human-specific gene networks, but in cell populations that are unrelated to brain development and function [8, 9].

To overcome the limitations of animal models and available human tissues, we differentiated WBS patient-specific induced pluripotent stem cells (iPSC) into cortical neurons for modeling the neuronal phenotype of WBS. Our analyses suggest that all layers of the cerebral cortex were represented by our population of iPSC derived neurons and the cells behaved like traditionally derived neurons, indicating successful differentiation. WBS-derived neurons showed altered action potential repolarization times when compared to control neurons, as determined through electrophysiology analysis, suggesting a defect in potassium channel conductance. We also examined gene expression and identified differentially expressed genes that implicate alterations in neurotransmitter receptors, synapse assembly and potassium channel complexes.

Recent iPSC modeling of WBS has focussed on expression in stem cells [10]. To our knowledge, our study is the first description of WBS phenotypes in patient-derived neurons, and is one of the first attempts to model WBS neurological phenotypes *in vitro* from any species. Our results suggest that patient-derived iPSC neurons represent a valid alternative for studying the molecular basis of complex neurological phenotypes, and provide evidence for altered function in WBS neurons.

## Results and discussion

### Differentiated neurons from WBS-iPSC display neuronal phenotypes

Previously, skin fibroblasts from a WBS patient were successfully reprogrammed to iPSCs and characterized for pluripotency and normal karyotype. Furthermore, these iPSC lines were differentiated into smooth muscle cells and the vascular disease phenotype was rescued by rapamycin [11]. Kinnear et al. also confirmed that the deletion spanned, at minimum, the sequence between FKBP6 and CLIP2. The patient displayed characteristic cardiac abnormalities in infancy, and later developed neurological phenotypes including mild global developmental delay involving fine motor and language skills noted since 2 years of age as well as clinical autism. However, gene testing for FMR1 CGG repeats for Fragile X syndrome was negative (data not shown). The patient receives occupational, physiotherapy, speech and behavior therapy. Here we used the same WBS iPSC lines for direct differentiation into cortical neurons, to gain insights into the neurological phenotype associated with WBS.

**Fig. 1:**
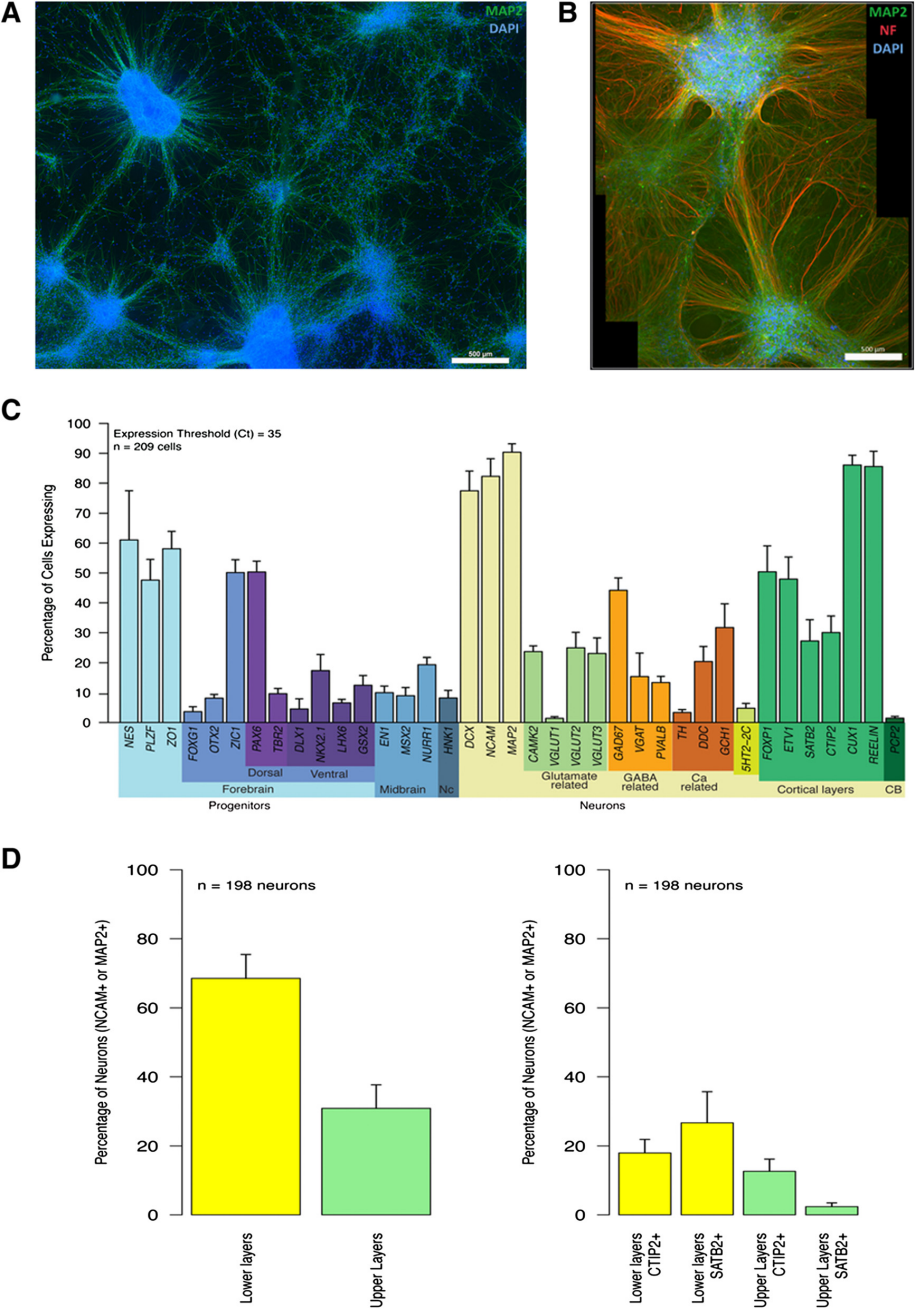
Differentiated WBS-neurons represent all cortical layers of the brain and show normal neuronal morphology. Six week WBS A neuronal culture stained with dendritic marker (MAP2) alone (**a**) and together with neurofilament (NF-axonal marker) (**b**). **c** Fluidigm single cell qRT-PCR analysis to characterize the neurons and efficiency of the differentiation protocol. The depicted graph shows an average value of all WBS and WT neurons. **d** Distribution of generated neurons for various brain cortical layers based on marker gene expression profiles

Three WBS (A, B, and I) lines and a WT iPSC line Δ3-4-hiPS #37 [12] were directly differentiated into cortical neurons with slight modifications from the previously published method [13]. We performed simultaneous differentiations of all 4 lines with triplicate wells tested for every assay including Fluidigm single cell expression, immunostaining, electrophysiology, and genome-wide expression profiling. In brief (Additional file 1: Figure S1A), cellular aggregates were plated to form neural rosettes (Additional file 1: Figure S1B) that were isolated and grown as Nestin + neural progenitor cells (Additional file 1: Figure S1C, E). The neural progenitors were induced to form neurons that were shown to have characteristic morphology (Additional file 1: Figure S1D). After 6–8 weeks of maturation in culture, neurons were immunostained and shown to have MAP2+ dendrites and NF+ axons (Fig. 1a,b).

To characterize and determine the efficiency of the differentiation protocol, a total of 209 single live cells (from all 4 lines (WT and WBS A, B, I) were sorted, and RNA extracted for quantitative reverse transcriptase real-time PCR (qRT-PCR) analyses using a Fluidigm array of 48 genes that mark different progenitors or neuronal subtypes [14, 15]. This single cell analysis indicated that the protocol was efficient in giving rise to neurons, with 80–90 % of cells expressing the mature neuronal markers DCX, NCAM1, and MAP2 (Fig. 1c). These neurons also expressed markers from upper and lower cortical layers, and the subtypes of cells identified include glutamatergic and GABA-ergic neurons (Fig. 1c,d). Similar to the findings of Pasca et al. some promiscuous expression of progenitor markers is detected in these cells (Fig. 1c) [14]. Nevertheless, the cellular morphology and neuronal marker expression were consistent with the efficient production of mixed cortical neurons in vitro and no substantial differences in marker expression were observed in neurons derived from the four iPSC lines.

### WBS-derived iPSC neurons show prolonged decay of action potentials, and impaired voltage-gated K^+^ channel currents

**Fig. 2:**
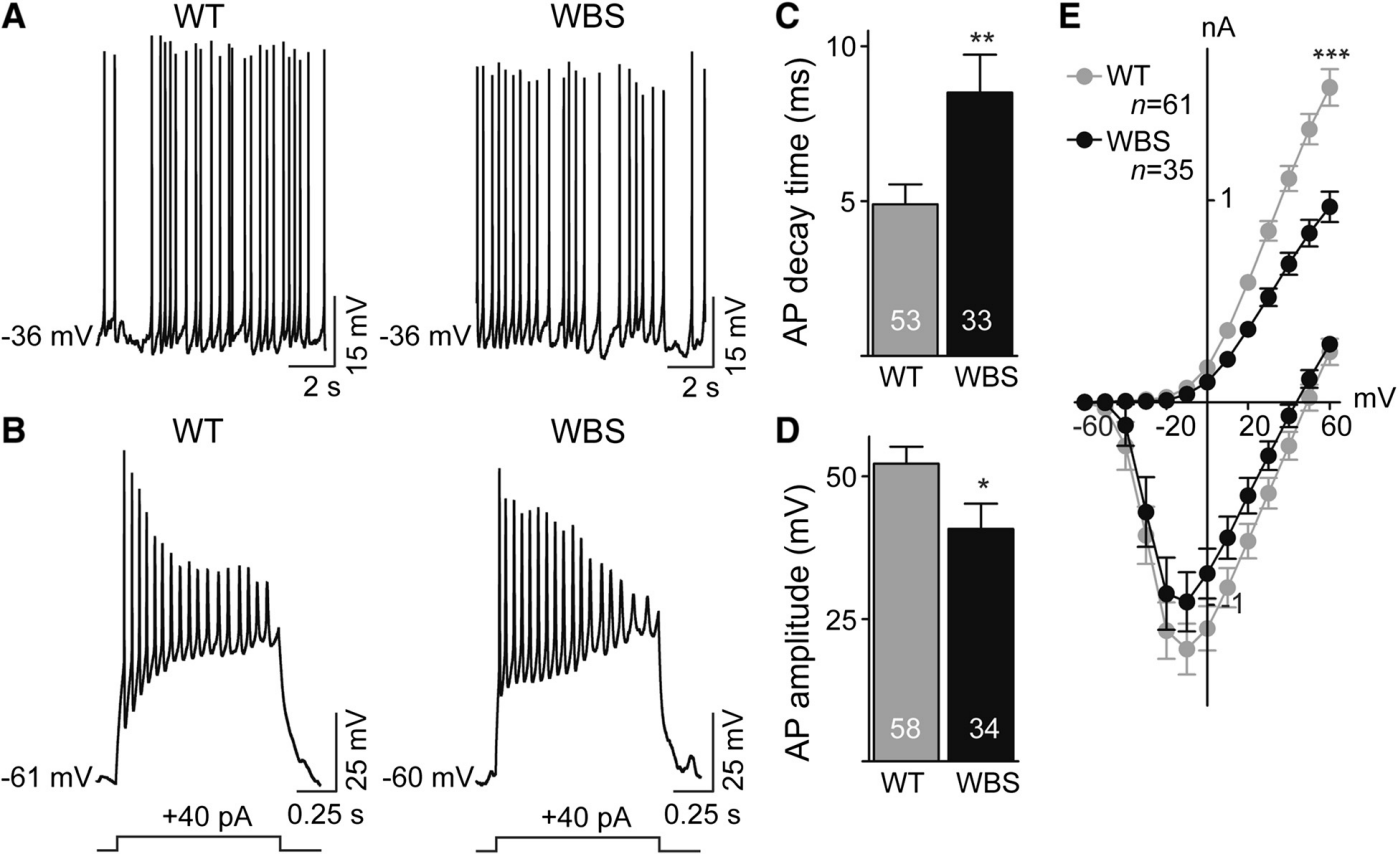
WBS-neurons display prolonged decay of evoked action potentials and decreased voltage-gated K^+^ currents. **a** Spontaneous action potentials were identified in WT- (Left) and WBS- (Right) neurons. **b** Representative traces show membrane potentials responding to injection of current step in WT- (Left) and WBS- (Right) neurons. **c**-**d** Histograms represent average decay time and amplitude of evoked action potentials for all cell lines of WT- and WBS-neurons. “*n*” represents number of neurons recorded. *P < 0.05, **P < 0.01. **e** A plot showing average current–voltage relationships of voltage-gated Na^+^ and K^+^ currents between WT- and WBS-neurons. ***P < 0.001

To determine whether there are electrophysiological alterations in WBS neurons, we obtained whole-cell patch-clamp recordings from neurons in three WBS lines (A, B, I), and in three WT lines – the line described above plus two additional lines from a different unaffected control individual. We found that the electrophysiological results from the original WT line and the two additional WT lines were not substantially different (not illustrated) and therefore, the data from these three lines were pooled as WT for the comparisons with the WBS lines. Both WT- and WBS-iPSC neurons can generate spontaneous (Fig. 2a) and/or evoked action potentials (Fig. 2b) that were blocked (Additional file 2: Figure. S2A and B) by the voltage-gated Na^+^ channel inhibitor tetrodotoxin (TTX; 0.5 μM). We found that the resting membrane potential in WBS neurons was not different from those in WT-neurons (Additional file 2: Figure S2F), but the input resistance was higher in WBS-neurons, compared with WT-neurons (Additional file 2: Figure S2E). Action potential characteristics of WBS-neurons and those from WT-controls were compared in recordings from over 30 neurons of each genotype. There was no significant difference observed in rise time (Additional file 2: Figure S2D) of the evoked action potentials between WT- and WBS-neurons. In contrast, WBS neurons displayed deficits in action potential repolarization: decay time (Fig. 2c) was significantly longer in WBS as compared with WT-neurons. In addition, the amplitude of the action potentials was smaller in WBS-neurons, compared with WT-neurons (Fig. 2d). We found a significant decrease in voltage-gated K^+^ currents in WBS-neurons, the channels responsible for repolarization [16], recorded under voltage-clamp conditions, compared with WT-neurons (Fig. 2e). However, voltage-gated Na^+^ currents in WBS neurons were not different from those in WT neurons (Fig. 2e). Both WT- and WBS-lines displayed spontaneous synaptic activity (Additional file 2: Figure S2G and H). We characterized miniature excitatory synaptic currents (mEPSC) and found no significant difference in amplitude (Additional file 2: Figure S2I) or frequency (Additional file 2: Figure S2J) between WBS- and WT-neurons. Thus, WBS neurons appear to have normal unitary excitatory synaptic transmission and the major electrophysiological defects are in action potential repolarization and voltage-gated K^+^ currents.

### Neuronal genes in the WBS deleted region are downregulated in WBS neurons

WBS is caused by the hemizygous deletion of multiple genes at 7q11.23, but it is not known which of these genes are misexpressed in WBS-neurons, or whether they control other target genes that could participate in the neuronal phenotypes described here. In order to determine whether the observed electrophysiology phenotype could be attributed to changes in gene expression, neuronal cultures generated from each of the three WBS-iPSC lines (A, B, and I) were compared to the control neuronal line on an Illumina HT-12 v4 Beadchip (GSE67535). Each of these samples contained RNA pooled from 3 wells of neurons.

To determine whether gene expression data from the control sample (WT) is representative of other wild-type samples, we integrated our data with expression data produced on the same platform (Illumina HumanHT12-v4) from wild-type iPSC-derived neurons in Stein et al. [17] samples Gage-1, Gage-2, Gage-3, Gage-4, Gage-5, Gage-6 (Additional file 3: Figure S3). Visual inspection of hierarchical clustering revealed that our control sample clustered with control samples from the Gage lab and not with WBS samples. Thus, our control sample was representative of the gene expression pattern in wild-type iPSC-derived neurons for downstream analyses.

**Fig. 3:**
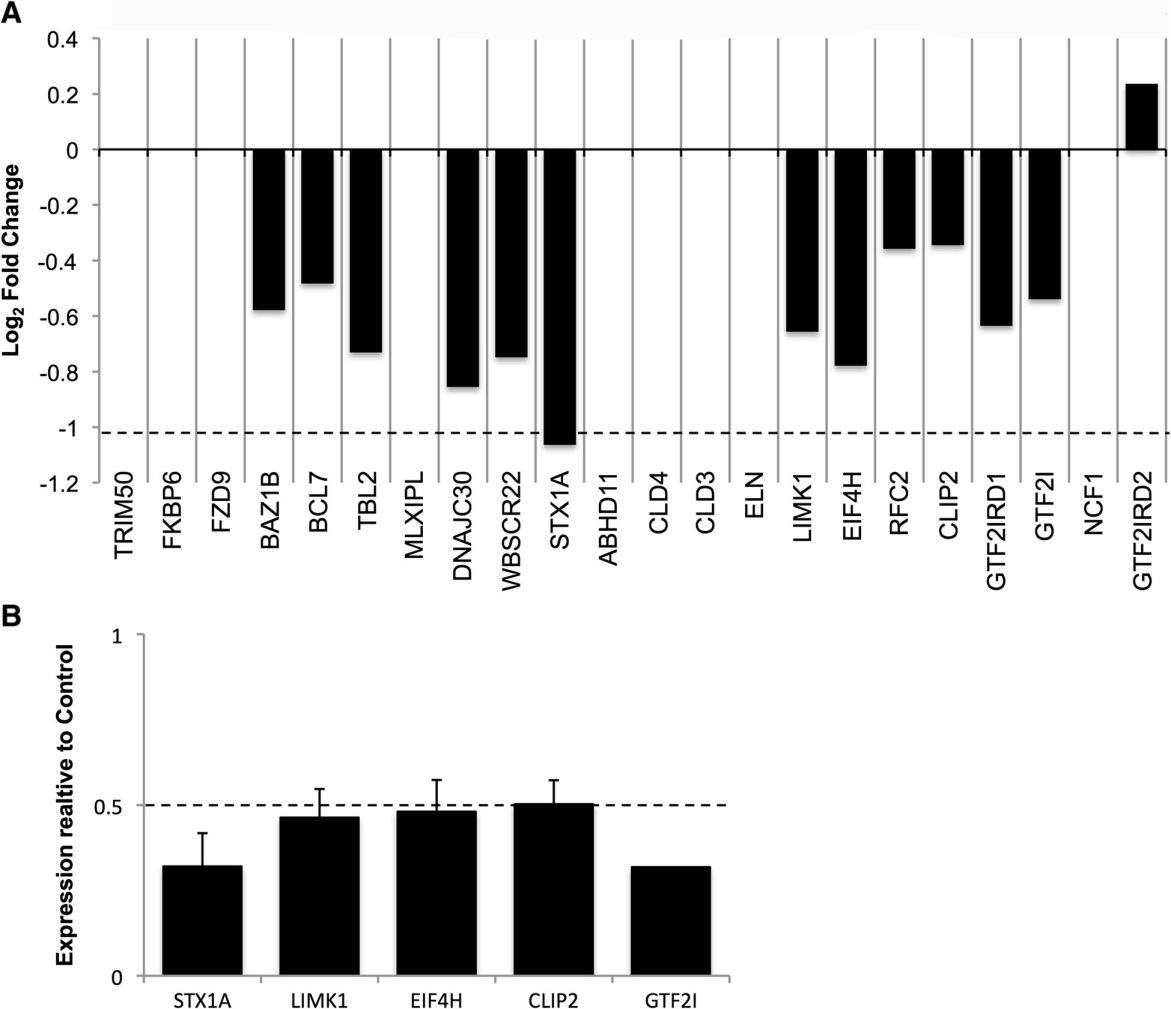
Genes within the hemizygous WBS deletion show reduced expression in differentiated iPSC neurons. The primers used for validation are described in Additional file 5: Table S1. **a** A bar graph of the neuronally expressed genes at 7q11.23 show the log2 fold change expression of WBS- vs. WT-iPSC neurons, as determined through microarray analysis. Genes with no change in expression were below the array detection threshold (average signal ≥100). All expressed genes that were confirmed to be deleted through MLPA were found to be downregulated [11]. A log2 fold change of -1 corresponds with half the expression of the controls as indicated by the dashed line. **b** qRT-PCR of five neuronally expressed genes was used to verify the array results. Expression was normalized to *HMBS*, and an averaged fold change of each WBS neuronal line (*n* = 3) is presented relative to the WT control. All five genes were expressed at less than half the level of expression in WT iPSC-derived neurons. Data are shown as mean ± SEM

Examination of genes from within the WBS commonly deleted region at 7q11.23 showed that most genes were expressed in iPSC-derived neurons, and were downregulated in the WBS neurons compared to WT neurons (Fig. 3a). Those genes that did not meet the expression threshold on the array (Detection *P*-value <0.01) were excluded from further analysis. To validate the changes detected in the array, qRT-PCR was used to determine the expression levels of five neuronally expressed genes (STX1A, LIMK1, EIF4H, CLIP2, GTF2I) from the WBS region, four of which have been linked to the neurological profile [18–23]. All five genes were expressed below 50 % of the level of expression in the WT neurons, indicating that there was no transcriptional compensation for the loss of one allele (Fig. 3b).

### Global gene expression is altered in WBS neurons

**Fig. 4:**
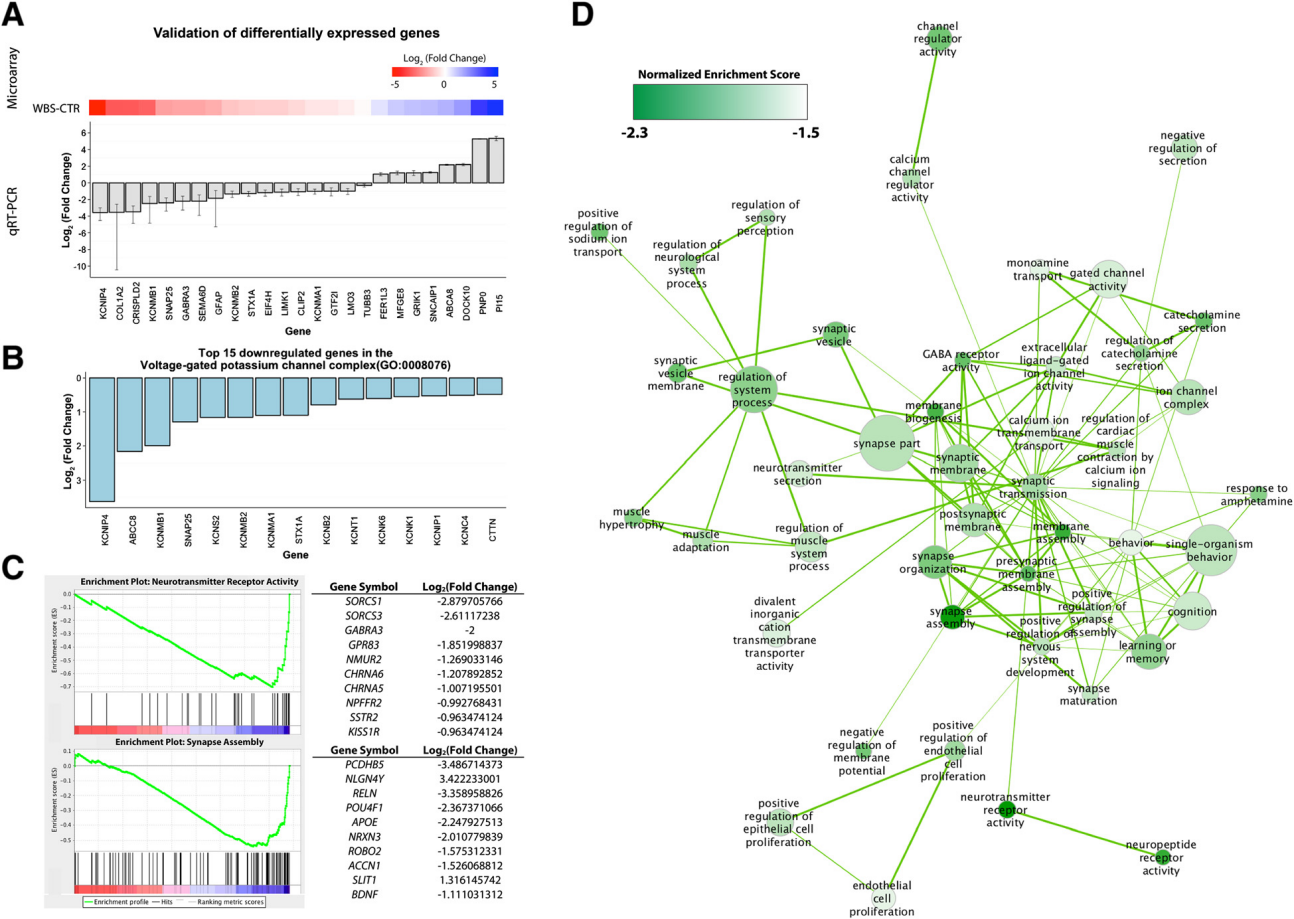
qRT-PCR Validation and gene set enrichment analysis. **a** Twenty-five differentially expressed genes were chosen for validation using qRT-PCR. Expression values from each WBS iPSC neuronal line (*n* = 3) were normalized to *HMBS* and *TBP*, and are presented as average fold change relative to WT control. All 25 genes showed altered expression in the same direction as identified on the array; the corresponding heatmap shows the log2 fold change in expression values from the array, with red indicating lower and blue indicating higher expression in WBS samples. Data are shown as mean ± SEM. **b** Log2 fold change of the top 15 downregulated genes in the voltage-gated potassium channel complex (GO:0008076) from the microarray. **c** Enrichment Plots of the two most highly enriched gene sets in the dataset. The top 10 genes in each gene set, ranked by absolute fold change, are shown in the corresponding tables (Additional file 6: Table S2, Additional file 7: Table S3, Additional file 8: Table S4 and Additional file 9: Table S5). **d** Enriched synaptic gene sets are highly interconnected. Enrichment Map [25] in Cytoscape was used to visualize overlap between enriched gene sets (FDR <0.1) as a network of interconnected nodes. The size of each node corresponds to the size of the gene set. The node color corresponds to the normalized enrichment score, with a darker green corresponding to stronger negative enrichment (lower expression in WBS relative to WT). The size of the edges between nodes corresponds to the number of genes the gene sets share in common. The largest interconnected network of nodes, which was comprised of gene sets governing synaptic function, is shown (46 out of a total 136 nodes). The full enrichment map is shown in Additional file 4: Figure S4

We chose 25 neuronally-expressed genes for validation using qRT-PCR. All were shown to have altered expression in the same direction as seen on the array (Fig. 4a). To investigate possible mechanisms of voltage-gated potassium channel dysfunction, as revealed by electrophysiology, we examined expression of genes in the voltage-gated potassium channel complex gene set (Gene Ontology gene set GO:0008076). We found that the vast majority of them were down-regulated (Fig 4b), including qRT-PCR validated reductions in KCNIP4, KCNMB1, KCNMB2, KCNMA1 in addition to WBS-deletion region gene STX1A and SNAP25 (Fig. 4b). To determine which functions were perturbed in WBS neurons, we performed a continuous gene set enrichment analysis [24] with gene sets from the gene ontology database. We ranked differentially expressed probes by the t-statistic and tested which gene sets were overrepresented among top-ranked (i.e. upregulated in WBS) or bottom-ranked (i.e. downregulated in WBS) genes (Additional file 6: Table S2, Additional file 7: Table S3, Additional file 8: Table S4 and Additional file 9: Table S5). Out of 5370 gene sets, 136 were enriched in WBS samples at a false discovery rate of 0.1. Strikingly, all 136 gene sets had negative enrichment scores, suggesting that they are enriched among bottom-ranking genes. The top negatively enriched gene sets were ‘neurotransmitter receptor activity’ (GO:0030594) and ‘synapse assembly’ (GO:0007416) (Fig. 4c).

To discover functional themes connecting the 136 gene sets, we visualized their overlap using the Enrichment Map plugin in Cytoscape (Additional file 4: Figure S4). Here, gene sets are visualized as a network of interconnected nodes based on the number of genes they have in common [25]. The largest connected network was found for gene sets governing synaptic function (46 gene sets total, Fig. 4d). This analysis suggested that ion channels, presynaptic function and GABA receptor function were particularly affected in WBS neurons (Fig. 4a). In addition, we found a network of 7 gene sets governing extracellular matrix function (ECM), with several gene sets showing particularly strong enrichment (Additional file 4: Figure S4), which may be analogous to the known role of ELN insufficiency in the cardiovascular WBS phenotype.

The affected gene sets we discovered in WBS neurons, in part, match those found in a recent gene expression study on WBS iPSCs and NPCs by Adamo et al. [10]. In common, we found enrichment of gene sets relating to synapse assembly, synaptic transmission and ECM organization. In addition, we also found two networks of gene sets governing heart and renal development (Additional file 4: Figure S4) also observed by Adamo et al. [10] and known to be perturbed in WBS.

Of those transcripts identified as belonging to significantly enriched gene sets, many encoded ion channels. Using qRT-PCR, we validated the upregulation of glutamate receptor subunit GRIK1 and the downregulation of GABA receptor subunit GABRA3. Dysregulation of either gene would suggest perturbations in neuronal signalling and function; interestingly, decreased expression of GABRA3 has been found in brains of patients with autism spectrum disorder [26, 27] and a truncating mutation has been found in a male with ASD [28].

In addition, we observed downregulation of transcripts for voltage-gated Na^+^ and K^+^ channels in WBS neurons. Downregulation of SCN3B and SCN2A Na^+^ channel subunits may explain the observed smaller amplitude of action potentials (Fig. 2d). The prolonged decay time observed in WBS neurons could be caused by altered function of voltage-gated K^+^ channels (Fig. 2e). This could be attributed to the observed downregulation of one, or a combination, of KCNIP4 (voltage-gated potassium channel gene), KCNMB1, KCNMB2 and KCNMA1 (calcium-activated potassium channel genes). However, we cannot exclude the possibility that the decrease of voltage-gated K^+^ current was due to the altered expression of other proteins directly and/or indirectly regulating K^+^ channels.

Voltage-gated K^+^ channels are broadly expressed in various types of neurons in the central nervous system and play critical roles in regulating neuronal excitability. Mutations of the proteins mediating the voltage-gated K^+^ currents can cause cellular dysfunction and diseases, and the channels have been used as therapeutic targets [16]. Decreased expression of gene KCTD7, a progressive myoclonus epilepsy gene encoding voltage-gated K^+^ channel tetramerisation domain containing 7, has been reported in WBS patients [29]. Additional reports have indicated that WBS patients suffer from progressive hearing loss of unknown cause [30–32]. Mutations in the proteins mediating voltage-gated KCNQ4 channel, a subtype of KV7/KCNQ/M channels, can lead to progressive hearing loss [33]. Prolongation of action potential repolarization in our present study, due to the decrease in voltage-gated K^+^ currents in WBS neurons, may perturb temporal and spatial integration for input at the somata, and thus alter the neuronal firing output.

At synapses, we found that unitary mEPSCs are not altered in WBS neurons (Additional file 2: Figure S2I,J) indicating that quantal size is not different. Moreover, mEPSC frequency was not altered in WBS neurons indicating that action potential-independent transmitter release is not affected. However, the prolongation of action potential repolarization which we observed (Fig. 2) may facilitate Ca^2+^ influx and thereby Ca^2+^-dependent transmitter release [34]. Thus, action potential-dependent transmission might be increased at WBS neuronal by increasing quantal content.

The gene sets downregulated in WBS neurons extend our understanding of WBS-specific alterations in neuronal gene expression. There is significant overlap among these gene sets with those reported in the gene expression study on WBS iPSCs and NPCs by Adamo et al. [10]. Common gene sets belong to synapse assembly, synaptic transmission, ECM organization and two networks of gene sets governing heart and renal function that are known to be perturbed in WBS (Additional file 4: Figure S4). The significance of these heart and renal networks to neuronal function is currently unclear and we cannot rule out the possibility that their appearance is due to the presence of contaminating disease-relevant cell types in our differentiations. Novel to WBS neuronal function, we have found a profound reduction of transcript levels of voltage-gated potassium channel genes (Fig 4b), as well as neurotransmitter receptor genes (Fig 4c). These changes may affect electrophysiological properties, as well as alter neurotransmission in WBS neurons. The prolongation of action potential repolarization in WBS neurons in our experiments provides support for the former. Taken together, our results extend the findings of Adamo et al. [10] to include abnormalities in neuronal gene expression.

## Conclusions

Our findings show that WBS neurons derived from patient iPSCs have dysregulated expression of genes including those encoding many ion channels that lie outside the WBS deletion. The expression profile is consistent with the observed electrophysiological defects with prolonged action potential repolarization resulting from a deficit in voltage-activated K^+^ currents. The observed alterations in K^+^ currents and action potential repolarization are predicted to dramatically alter integration, output and action potential-dependent transmission in networks of WBS neurons. Such altered cellular and network functioning may be a critical determinant underlying deficits in visuospatial construction and cognitive ability in patients with WBS.

## Methods

### WBS iPS cell line derivation and directed neuronal differentiation

The derivation and pluripotency characterization of WBS iPS cell lines has been reported previously [11]. Neuronal differentiations were carried out as described previously [13] but with minor modifications, which are described here in detail. Briefly, WBS (A, B and I hiPSC lines) and WT iPSC line (Δ3-4-hiPS #37) [12] were dissociated to form cellular aggregates and cultured in suspension on low cluster plates with media changes everyday in Knockout DMEM containing knockout serum replacement, GlutaMAX-1, non-essential amino acids, 2-marcaptoethanol and supplemented with bFGF (10 ng/ml), SB431542 (10 μM) and Dorsomorphin (2 μM). The media was switched to neural induction media on day 4 containing DMEM-F12, N2, non-essential amino acids, Heparin (2 μg/ml), bFGF (10 ng/ml), SB431542 (10 μM) and Dorsomorphin (2 μM). On day 6, the cellular aggregates were seeded onto polyornithin/laminin coated dishes in neural induction media without SB431542. The cells were fed with fresh media every second day till day 17. On the 17th day of differentiation, the neural rosettes were manually dissected and plated onto polyornithin and laminin coated plates to obtain secondary rosettes. The media was changed every second day with neural induction media without SB431542 and Dorsomorphin. On day 25 of differentiation, the secondary rosettes were manually harvested from the plates and spun down for 2 mins at 600 rpm (69 g) in a swing bucket tabletop centrifuge. The cell pellet was resuspended in 1 ml acutase and incubated for 10 min at room temperature. 1 ml of neural precursor media (DMEM F12, N2, B27 + RA, laminin, non-essential amino acids, heparin, bFGF) was added and cells were triturated gently to break apart the rosettes and obtain single cells. Cells were pelleted at 600 rpm (69 g) for 2 mins, resuspend in 2 ml of neural precursor media, and plated onto polyornithin/laminin coated plates. For neuronal differentiations, glass cover slips were coated with polyornithin and laminin and placed into 24 well plates. The neural precursor cells were plated at a density of 5 × 10^4^ cells per well in complete neural differentiation media (Neurobasal, N2, non-essential amino acids, BDNF (10 ng/ml), GDNF (10 ng/ml), IGF-1 (10 ng/ml), B27 (without RA), cAMP (1 μM), Ascorbic acid (200 ng/ml), Laminin (1 μg/ml). The media was changed every second day for 6–8 weeks. The protocol was repeated at least three independent times starting from hES/iPS cells. The generation and description of two additional human WT iPSC lines from a different unaffected control individual, and their neuronal differentiation was described in Zhang et al. (submitted).

### Characterization of neurons using single cell qRT-PCR and immunostaining

Three wells of each WT, WBS A, WBS B, and WBS I neuronal cultures (after 6 weeks of maturation) were dissociated with acutase and sorted based on propidium-iodide into 96-well qRT-PCR plates. The pre-amplification mix and primers for individual gene expression analysis and Fluidigm Dynamic Arrays were performed as described previously [14]. Fluidigm data analysis was performed using the R-Statistical analysis software (https://cran.r-project.org/), for which scripts are available upon request. Cells were identified based on expression of both GAPDH and 18S. A cell was defined as expressing a given marker if it exhibited detectable cDNA of the appropriate melting temperature during the course of a 35 cycle Fluidigm run. Cells expressing NCAM, MAP2 and DCX were classed as neurons. Further classification by cortical layer and neurotransmitter type was defined as follows: ETV1+ or FOXP1+, lower layer; ETV1- FOXP1- and any of CUX1+ SATB2+ CTIP2+ REELIN+, upper layer; GAD65+ GAD67+ VGAT+ CALB1+ CALB2+ or PVALB+, GABAergic; CAMK2+ VGLUT1+ VGLUT2+ or VGLUT3+, glutamatergic.

Immunostaining of neuronal cultures were done as described previously [12]. Antibodies against phosphorylated neurofilaments (SMI-31R) Covance (1:1000 dilution), and MAP2 (AB5622) Millipore (1:2000 dilution) were used to perform the immunostaining.

### Electrophysiology

Human iPSC-derived neurons after 6 weeks of culture were used for whole-cell patch-clamp recordings at room temperature as previously reported for mouse neurons [35]. An Axopatch 1-D amplifier (Molecular Devices) was used and the signals were low-pass filtered at 2 kHz. Micropipette electrodes were prepared from borosilicate capillary glass (World Precision Instruments, Inc., USA), using a P-87 pipette puller (Sutter Instrument Co., USA). The intracellular recording solutions consisted of (in mM): 144 K^+^-gluconate, 10 KCl, 10 HEPES, 2 EGTA, and 2 Mg^2+^-ATP, pH adjusted to 7.2 with KOH. The external recording solutions were composed of (in mM): 140 NaCl, 5.4 KCl, 1 MgCl_2_, 15 HEPES, 2 CaCl_2_, and 10 glucose, adjusted to pH 7.34 with NaOH. Voltage-gated ionic currents, under whole-cell voltage-clamp conditions, were elicited by depolarizing neuronal membrane potentials to a series of potentials from -60 mV to 60 mV for 400 ms, and voltage-gated K^+^ currents were calculated at the end of the depolarizing step. To evoke action potentials in iPS cell-derived neurons, a series of current steps from -5 pA to +50 pA, under whole-cell current-clamp conditions, were injected every 10 s, and action potential parameters were based on analysis of the first evoked action potentials.

Whole-cell voltage-clamp recording was performed on iPSC-derived neurons at the membrane potential of -60 mV for recording mEPSC with the external recording solutions (in mM, pH 7.35): 140 NaCl, 5.4 KCl, 1.3 CaCl_2_, 15 HEPES, 25 glucose, 0.0005 tetrodotoxin, 0.001 glycine; 0.01 bicuculline, and 0.01 strychnine. The internal solutions contained (in mM, pH 7.2): 137 CsF, 1.5 CsCl, 10 HEPES, 10 BAPTA, 4 Mg^2+^-ATP. The recordings were made at room temperature, and the mEPSC activities were analyzed off-line using Mini Analysis Program (Synaptosoft Inc, NJ, USA). Statistical analysis was performed using Student’s *t*-test. Data were displayed as mean ± SEM.

### Microarray analysis using Illumina HT-12 v4 Beadchip

Three wells of each WT, WBS A, WBS B, and WBS I differentiated neurons were pooled to extract RNA. 1 μg of RNA from each iPSC derived neuronal sample; WT, WBS A, WBS B, and WBS I; was submitted to The Centre for Applied Genomics at the Hospital for Sick Children, Toronto, for analysis on the Illumina HT-12 v4 Beadchip array (Illumina, San Diego, CA), which targets over 47,000 probes. Raw intensity values were exported and analyzed using the R limma package. To determine whether gene expression data from our control sample (WT) is representative of other wild-type samples, we integrated our data with expression data produced on the same platform (Illumina HumanHT12-v4) from wild-type iPSC-derived neurons in Stein et al. [16] samples Gage-1, Gage-2, Gage-3, Gage-4, Gage-5, Gage-6. Supplementary data containing background-corrected unnormalized probe intensity values was downloaded from the gene expression omnibus (GEO Accession: GSE57595). This data was merged with unnormalized, background-corrected (nec function in limma [36]) values from our dataset. The merged dataset was log2-transformed, quantile normalized and batch-corrected using the R package ComBat.

To compare gene expression between our WT sample and the three WBS samples, the probe IDs were converted to nuIDs to improve annotation consistency. The neqc function [36] was used for background correction and quantile normalization. The data were log2 transformed and probes with detection *p*-values >0.01 in all samples were removed for downstream analyses (22134 probes remaining out of 47320). To perform gene set enrichment analysis, all probes were ranked by the t-statistic produced by an empirical Bayes statistics (eBayes) of the resulting linear model (lmfit) of the microarray. We used gene sets from the Gene Ontology: Biological Process database and filtered out datasets that were overly large (>900 genes) or overly small (<15 genes). Gene sets enriched at a false discovery rate (FDR) of <0.1 were used to construct an Enrichment Map [25] in Cytoscape [37] to visualize gene set interconnectedness and identify perturbed cellular functions.

### RNA extraction, cDNA synthesis, and qRT-PCR analysis

500 ng of RNA was converted to cDNA using Superscript III Reverse Transcriptase according to manufacturer guidelines (Life Technologies, Burlington, Canada). The samples were diluted 1:100, and quantitative real-time PCR (qRT-PCR) was used to validate hemizygosity of the WBS deletion region, and differentially expressed genes (Power SYBR Green PCR Master Mix, Life Technologies). The primers used for validation are described in Additional file 5: Table S1. qRT-PCR analysis was carried out on a ViiA 7 Real-Time PCR System (Life Technologies), and expression values were determined by interpolating a standard curve derived from human brain total RNA (Clontech, Mountain View, CA) as previously described [38]. Expression values from each of the three WBS iPSC neuronal lines were normalized to the housekeeping genes HMBS and TBP, and are presented as fold change relative to the WT control.

## Additional files

Additional file 1: Figure S1. Neuronal differentiation protocol and marker characterization. Outline of the differentiation protocol (A). Brightfield images of (day 17) neural rosettes (B), Neural precursor cells six days post plating (passage 1) from rosettes (C) and 4 week old Neurons (D) derived from WBS-iPS cells. Immunostaining of WBS-iPS cell derived (passage 10) neural precursor cells with Nestin (neural stem cell marker) and Dapi (blue-nuclear stain) (E). Scale bars: 100 μm (B,E), 200 μm (C,D). (TIF 33780 kb) Additional file 2: Figure S2. Action potentials are sensitive to tetrodotoxin. Both spontaneous (A) and evoked (B) action potentials were blocked by the application of TTX (0.5 μM) in WT-neurons. Bar graphs showing the half-duration (C) and rise time (D) of evoked action potentials in WT-neurons compared with WBS-neurons. Bar graphs showing average input resistance (E) and resting membrane potential (F) in WT-neurons compared with WBS-neurons, **P* < 0.05. Spontaneous mEPSC activities were recorded in WT- (G) and WBS- (H) neurons. Histograms showing amplitude (I) and frequency (J) of mEPSC in WT-neurons compared with WBS-neurons. (TIF 600 kb) Additional file 3: Figure S3. Hierarchical clustering plots of wild type neurons. Hierarchical clustering by differentially expressed genes using integrated microarray data. Microarray data from our study (WT and WBS samples: A, B, I) was integrated with expression data from wild-type iPSC-derived neurons run on the same Illumina HumanHT-12 v4 microarray from [17] (Samples Gage-1, Gage-2, Gage-3, Gage-4, Gage-5, Gage-6). Unnormalized data was downloaded from GEO (accession number GSE57595), merged with our unnormalized data, log2-transformed, quantile normalized, and corrected for batch effect using ComBat. Hierarchical clustering of differentially expressed genes shows that the WT sample from our study is representative of other wild-type samples. (TIF 54 kb) Additional file 4: Figure S4. Enrichment Map of all 136 enriched gene sets in the microarray data sets at FDR <0.1. Enrichment Map [25] in Cytoscape was used to visualize overlap between enriched gene sets (FDR <0.1) as a network of interconnected nodes. The size of each node corresponds to the size of the gene set. The node color corresponds to the normalized enrichment score, with a darker green corresponding to stronger negative enrichment (lower expression in WBS relative to WT). The size of the edges between nodes corresponds to the number of genes the gene sets share in common. (TIF 1360 kb) Additional file 5: Table S1. Primers used for quantitative real-time PCR validation of differentially expressed genes. (XLSX 54 kb) Additional file 6: Table S2. Table of negatively enriched gene sets. (XLSB 195 kb) Additional file 7: Table S3. Table of positively enriched gene sets. (XLSB 188 kb) Additional file 8: Table S4. Detailed tables for the top 20 negatively and positively enriched gene sets, sorted in order of decreasing absolute fold change. (XLS 308 kb) Additional file 9: Table S5. Table of differentially expressed genes used for ranking genes for GSEA. (XLS 4906 kb)

## Abbreviations

ADHD: Attention deficit, hyperactivity disorder
ECM: Extracellular matrix
FDR: False-discovery rate
iPSC: Induced pluripotent stem cell
mEPSC: miniature excitatory postsynaptic current
qRT-PCR: quantitative reverse transcriptase real-time PCR
WBS: Williams-Beuren Syndrome
WT: Wild-type
TTX: Tetrodotoxin

## References

[1] Pober BR (2010). Williams-Beuren syndrome. N Engl J Med.

[2] Mervis CB, Velleman SL (2011). Children with Williams Syndrome: Language, Cognitive, and Behavioral Characteristics and their Implications for Intervention. Perspect Lang Learn Educ.

[3] Klein-Tasman BP, Mervis CB (2003). Distinctive personality characteristics of 8-, 9-, and 10-year-olds with Williams syndrome. Dev Neuropsychol.

[4] Morris CA (2010). The behavioral phenotype of Williams syndrome: A recognizable pattern of neurodevelopment. Am J Med Genet C: Semin Med Genet.

[5] Meyer-Lindenberg A, Kohn P, Mervis CB, Kippenhan JS, Olsen RK, Morris CA (2004). Neural basis of genetically determined visuospatial construction deficit in Williams syndrome. Neuron.

[6] Meyer-Lindenberg A, Hariri AR, Munoz KE, Mervis CB, Mattay VS, Morris CA (2005). Neural correlates of genetically abnormal social cognition in Williams syndrome. Nat Neurosci.

[7] Osborne LR (2010). Animal models of Williams syndrome. Am J Med Genet C: Semin Med Genet.

[8] Henrichsen CN, Csardi G, Zabot MT, Fusco C, Bergmann S, Merla G (2011). Using transcription modules to identify expression clusters perturbed in Williams-Beuren syndrome. PLoS Comput Biol.

[9] Antonell A, Vilardell M, Perez Jurado LA (2010). Transcriptome profile in Williams-Beuren syndrome lymphoblast cells reveals gene pathways implicated in glucose intolerance and visuospatial construction deficits. Hum Genet.

[10] Adamo A, Atashpaz S, Germain PL, Zanella M, D’Agostino G, Albertin V (2015). 7q11.23 dosage-dependent dysregulation in human pluripotent stem cells affects transcriptional programs in disease-relevant lineages. Nat Genet.

[11] Kinnear C, Chang WY, Khattak S, Hinek A, Thompson T, de Carvalho RD (2013). Modeling and rescue of the vascular phenotype of Williams-Beuren syndrome in patient induced pluripotent stem cells. Stem Cells Transl Med.

[12] Cheung AY, Horvath LM, Grafodatskaya D, Pasceri P, Weksberg R, Hotta A (2011). Isolation of MECP2-null Rett Syndrome patient hiPS cells and isogenic controls through X-chromosome inactivation. Hum Mol Genet.

[13] Brennand KJ, Simone A, Jou J, Gelboin-Burkhart C, Tran N, Sangar S (2011). Modelling schizophrenia using human induced pluripotent stem cells. Nature.

[14] Pasca SP, Portmann T, Voineagu I, Yazawa M, Shcheglovitov A, Pasca AM (2011). Using iPSC-derived neurons to uncover cellular phenotypes associated with Timothy syndrome. Nat Med.

[15] Djuric U, Cheung AY, Zhang W, Mok RS, Lai W, Piekna A (2015). MECP2e1 isoform mutation affects the form and function of neurons derived from Rett syndrome patient iPS cells. Neurobiol Dis.

[16] Wulff H, Castle NA, Pardo LA (2009). Voltage-gated potassium channels as therapeutic targets. Nat Rev Drug Discov.

[17] Stein JL, de la Torre-Ubieta L, Tian Y, Parikshak NN, Hernandez IA, Marchetto MC (2014). A quantitative framework to evaluate modeling of cortical development by neural stem cells. Neuron.

[18] Frangiskakis JM, Ewart AK, Morris CA, Mervis CB, Bertrand J, Robinson BF (1996). LIM-kinase1 hemizygosity implicated in impaired visuospatial constructive cognition. Cell.

[19] Hoogenraad CC, Koekkoek B, Akhmanova A, Krugers H, Dortland B, Miedema M (2002). Targeted mutation of Cyln2 in the Williams syndrome critical region links CLIP-115 haploinsufficiency to neurodevelopmental abnormalities in mice. Nat Genet.

[20] Morris CA, Mervis CB, Hobart HH, Gregg RG, Bertrand J, Ensing GJ (2003). GTF2I hemizygosity implicated in mental retardation in Williams syndrome: genotype-phenotype analysis of five families with deletions in the Williams syndrome region. Am J Med Genet A.

[21] Gao MC, Bellugi U, Dai L, Mills DL, Sobel EM, Lange K (2010). Intelligence in Williams Syndrome is related to STX1A, which encodes a component of the presynaptic SNARE complex. PLoS One.

[22] Sakurai T, Dorr NP, Takahashi N, McInnes LA, Elder GA, Buxbaum JD (2011). Haploinsufficiency of Gtf2i, a gene deleted in Williams Syndrome, leads to increases in social interactions. Autism Res.

[23] Antonell A, Del Campo M, Magano LF, Kaufmann L, de la Iglesia JM, Gallastegui F (2010). Partial 7q11.23 deletions further implicate GTF2I and GTF2IRD1 as the main genes responsible for the Williams-Beuren syndrome neurocognitive profile. J Med Genet.

[24] Subramanian A, Tamayo P, Mootha VK, Mukherjee S, Ebert BL, Gillette MA (2005). Gene set enrichment analysis: a knowledge-based approach for interpreting genome-wide expression profiles. Proc Natl Acad Sci U S A.

[25] Merico D, Isserlin R, Stueker O, Emili A, Bader GD (2010). Enrichment map: a network-based method for gene-set enrichment visualization and interpretation. PLoS One.

[26] Okazaki Y, Furuno M, Kasukawa T, Adachi J, Bono H, Kondo S (2002). Analysis of the mouse transcriptome based on functional annotation of 60,770 full-length cDNAs. Nature.

[27] Fatemi SH, Reutiman TJ, Folsom TD, Thuras PD (2009). GABA(A) receptor downregulation in brains of subjects with autism. J Autism Dev Disord.

[28] Piton A, Jouan L, Rochefort D, Dobrzeniecka S, Lachapelle K, Dion PA (2013). Analysis of the effects of rare variants on splicing identifies alterations in GABAA receptor genes in autism spectrum disorder individuals. Eur J Hum Genet.

[29] Merla G, Howald C, Henrichsen CN, Lyle R, Wyss C, Zabot MT (2006). Submicroscopic deletion in patients with Williams-Beuren syndrome influences expression levels of the nonhemizygous flanking genes. Am J Hum Genet.

[30] Marler JA, Sitcovsky JL, Mervis CB, Kistler DJ, Wightman FL (2010). Auditory function and hearing loss in children and adults with Williams syndrome: cochlear impairment in individuals with otherwise normal hearing. Am J Med Genet C: Semin Med Genet.

[31] Matsumoto N, Kitani R, Kalinec F (2011). Linking LIMK1 deficiency to hyperacusis and progressive hearing loss in individuals with Williams syndrome. Commun Integr Biol.

[32] Johnson LB, Comeau M, Clarke KD (2001). Hyperacusis in Williams syndrome. J Otolaryngol.

[33] Jentsch TJ (2000). Neuronal KCNQ potassium channels: physiology and role in disease. Nat Rev Neurosci.

[34] Yang YM, Wang LY (2006). Amplitude and kinetics of action potential-evoked Ca2+ current and its efficacy in triggering transmitter release at the developing calyx of Held synapse. J Neurosci.

[35] Farra N, Zhang WB, Pasceri P, Eubanks JH, Salter MW, Ellis J (2012). Rett syndrome induced pluripotent stem cell-derived neurons reveal novel neurophysiological alterations. Mol Psychiatry.

[36] Shi W, Oshlack A, Smyth GK (2010). Optimizing the noise versus bias trade-off for Illumina whole genome expression BeadChips. Nucleic Acids Res.

[37] Shannon P, Markiel A, Ozier O, Baliga NS, Wang JT, Ramage D (2003). Cytoscape: a software environment for integrated models of biomolecular interaction networks. Genome Res.

[38] O’Leary J, Osborne LR (2011). Global analysis of gene expression in the developing brain of Gtf2ird1 knockout mice. PLoS One.

