# Supplementary figures and images for: Human induced pluripotent stem cell derived neurons as a model for Williams-Beuren syndrome
Source: Mol Brain. 2015 Nov 24;8:77. doi: 10.1186/s13041-015-0168-0 (PMC4657290; doi:10.1186/s13041-015-0168-0)

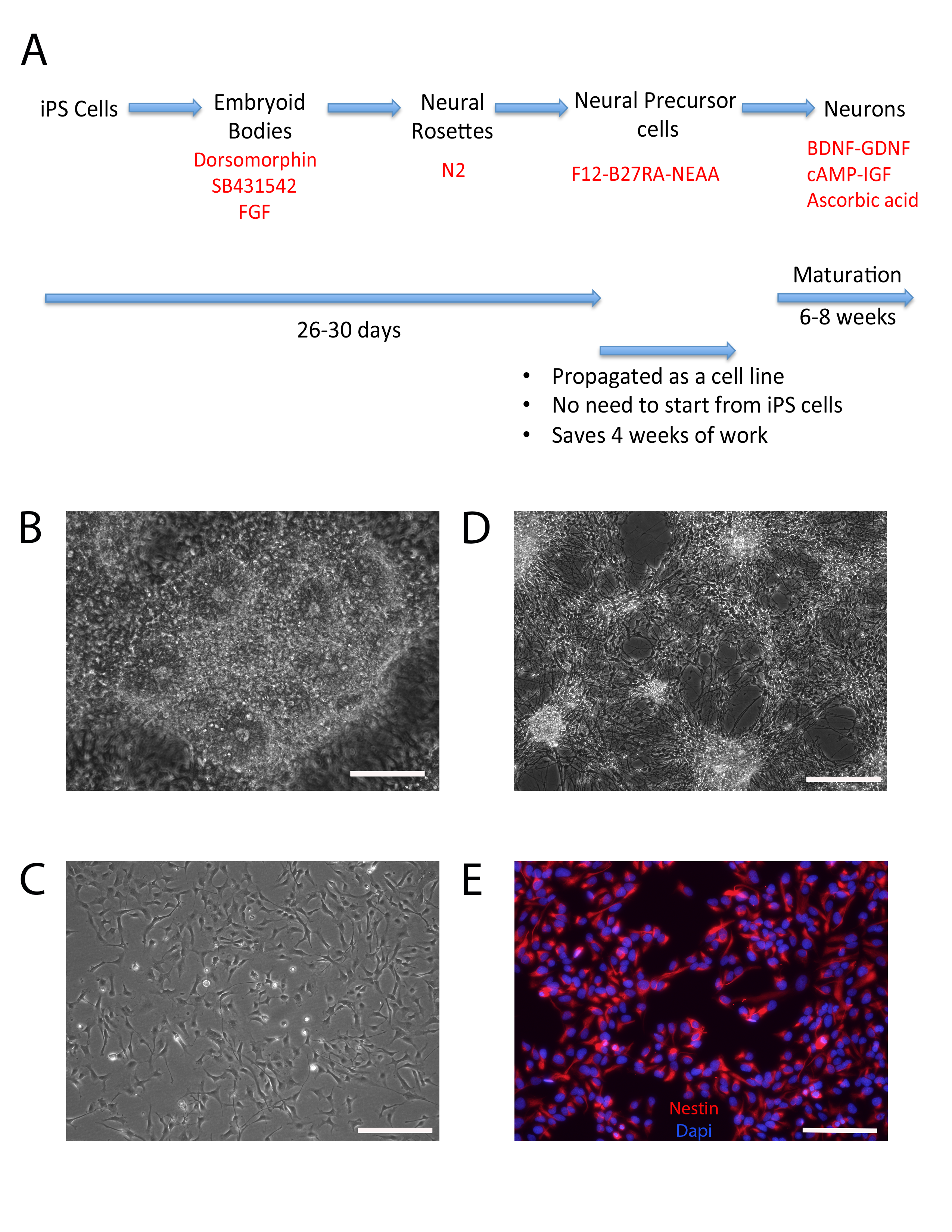

Supplement: Additional file 1: Figure S1. — Neuronal differentiation protocol and marker characterization. Outline of the differentiation protocol (A). Brightfield images of (day 17) neural rosettes (B), Neural precursor cells six days post plating (passage 1) from rosettes (C) and 4 week old Neurons (D) derived from WBS-iPS cells. Immunostaining of WBS-iPS cell derived (passage 10) neural precursor cells with Nestin (neural stem cell marker) and Dapi (blue-nuclear stain) (E). Scale bars: 100 μm (B,E), 200 μm (C,D). (TIF 33780 kb) [file 13041_2015_168_MOESM1_ESM.tif]

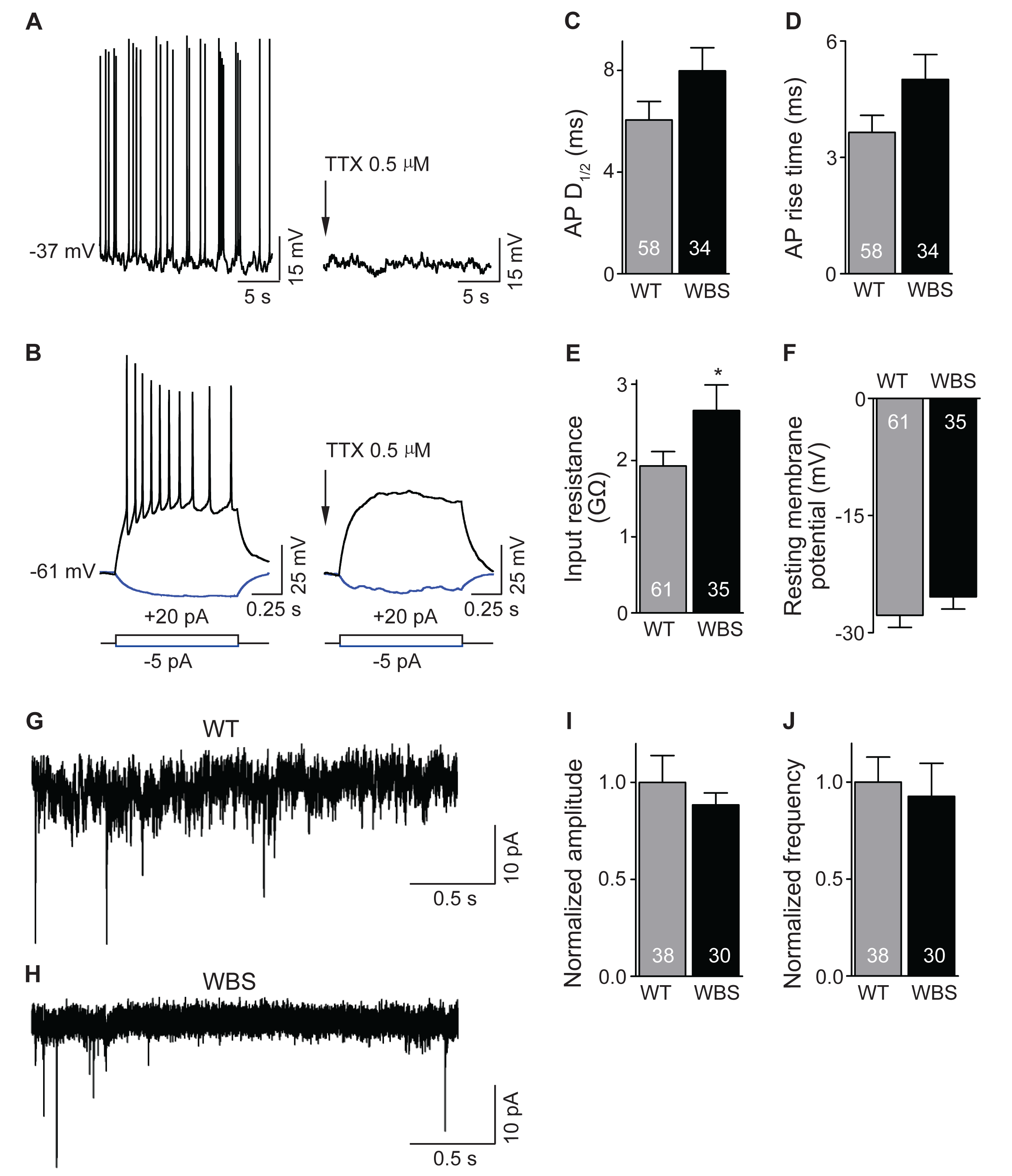

Supplement: Additional file 2: Figure S2. — Action potentials are sensitive to tetrodotoxin. Both spontaneous (A) and evoked (B) action potentials were blocked by the application of TTX (0.5 μM) in WT-neurons. Bar graphs showing the half-duration (C) and rise time (D) of evoked action potentials in WT-neurons compared with WBS-neurons. Bar graphs showing average input resistance (E) and resting membrane potential (F) in WT-neurons compared with WBS-neurons, *P < 0.05. Spontaneous mEPSC activities were recorded in WT- (G) and WBS- (H) neurons. Histograms showing amplitude (I) and frequency (J) of mEPSC in WT-neurons compared with WBS-neurons. (TIF 600 kb) [file 13041_2015_168_MOESM2_ESM.tif]

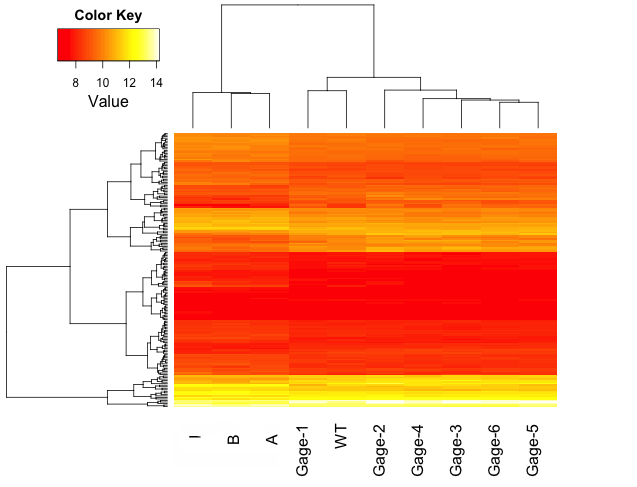

Supplement: Additional file 3: Figure S3. — Hierarchical clustering plots of wild type neurons. Hierarchical clustering by differentially expressed genes using integrated microarray data. Microarray data from our study (WT and WBS samples: A, B, I) was integrated with expression data from wild-type iPSC-derived neurons run on the same Illumina HumanHT-12 v4 microarray from [17] (Samples Gage-1, Gage-2, Gage-3, Gage-4, Gage-5, Gage-6). Unnormalized data was downloaded from GEO (accession number GSE57595), merged with our unnormalized data, log2-transformed, quantile normalized, and corrected for batch effect using ComBat. Hierarchical clustering of differentially expressed genes shows that the WT sample from our study is representative of other wild-type samples. (TIF 54 kb) [file 13041_2015_168_MOESM3_ESM.tif]

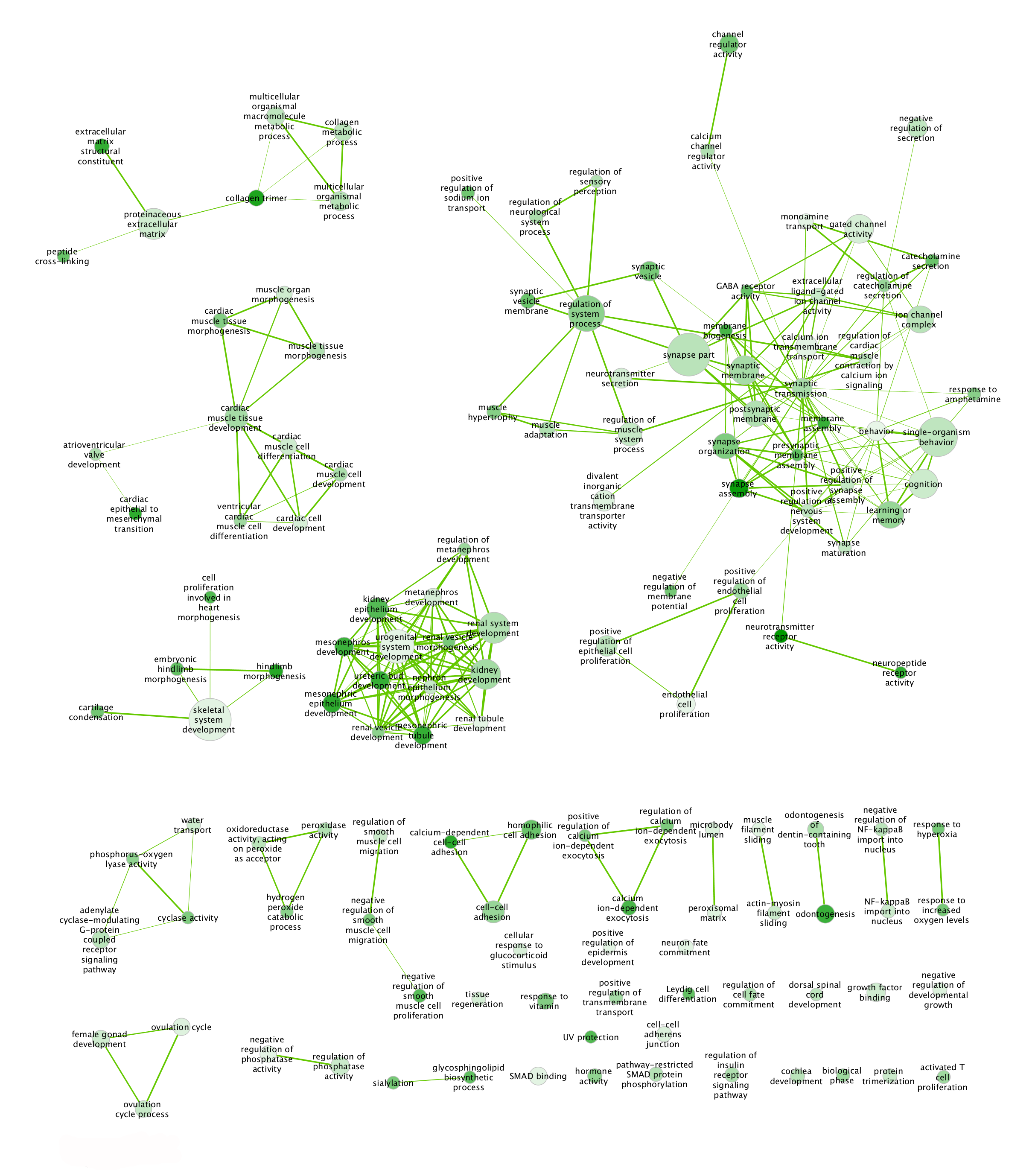

Supplement: Additional file 4: Figure S4. — Enrichment Map of all 136 enriched gene sets in the microarray data sets at FDR <0.1. Enrichment Map [25] in Cytoscape was used to visualize overlap between enriched gene sets (FDR <0.1) as a network of interconnected nodes. The size of each node corresponds to the size of the gene set. The node color corresponds to the normalized enrichment score, with a darker green corresponding to stronger negative enrichment (lower expression in WBS relative to WT). The size of the edges between nodes corresponds to the number of genes the gene sets share in common. (TIF 1360 kb) [file 13041_2015_168_MOESM4_ESM.tif]
